# Associations of MRI-visible perivascular spaces with longitudinal cognitive decline across the Alzheimer’s disease spectrum

**DOI:** 10.1186/s13195-022-01136-y

**Published:** 2022-12-13

**Authors:** Ming-Liang Wang, Qiao-Qiao Zou, Zheng Sun, Xiao-Er Wei, Peng-Yang Li, Xue Wu, Yue-Hua Li, for the Alzheimer’s Disease Neuroimaging Initiative

**Affiliations:** 1 Department of Radiology, Shanghai Sixth People’s Hospital Affiliated to Shanghai Jiao Tong University School of Medicine, No. 600, Yi Shan Road, Shanghai, 200233 China; 2 Division of Cardiology, Pauley Heart Center, Virginia Commonwealth University, Richmond, VA USA; 3 Institute for Global Health Sciences, University of California San Francisco, San Francisco, CA USA

**Keywords:** Perivascular spaces, MRI, Alzheimer’s disease, Cognitive impairment, Conversion

## Abstract

**Objective:**

To investigate the characteristics and associations of MRI-visible perivascular spaces (PVS) with clinical progression and longitudinal cognitive decline across the Alzheimer’s disease spectrum.

**Methods:**

We included 1429 participants (641 [44.86%] female) from the Alzheimer’s Disease Neuroimaging Initiative (ADNI) database. PVS number and grade in the centrum semiovale (CSO-PVS), basal ganglia (BG-PVS), and hippocampus (HP-PVS) were compared among the control (CN), mild cognitive impairment (MCI), and Alzheimer’s disease (AD) groups. PVS were tested as predictors of diagnostic progression (i.e., CN to MCI/AD or MCI to AD) and longitudinal changes in the 13-item Alzheimer’s Disease Assessment Scale-cognitive subscale (ADAS-Cog 13), Mini-Mental State Examination (MMSE), memory (ADNI-MEM), and executive function (ADNI-EF) using multiple linear regression, linear mixed-effects, and Cox proportional hazards modeling.

**Results:**

Compared with CN subjects, MCI and AD subjects had more CSO-PVS, both in number (*p* < 0.001) and grade (*p* < 0.001). However, there was no significant difference in BG-PVS and HP-PVS across the AD spectrum (*p* > 0.05). Individuals with moderate and frequent/severe CSO-PVS had a higher diagnostic conversion risk than individuals with no/mild CSO-PVS (log-rank *p* < 0.001 for all) in the combined CN and MCI group. Further Cox regression analyses revealed that moderate and frequent/severe CSO-PVS were associated with a higher risk of diagnostic conversion (HR = 2.007, 95% CI = 1.382–2.914, *p* < 0.001; HR = 2.676, 95% CI = 1.830–3.911, *p* < 0.001, respectively). A higher CSO-PVS number was associated with baseline cognitive performance and longitudinal cognitive decline in all cognitive tests (*p* < 0.05 for all).

**Conclusions:**

CSO-PVS were more common in MCI and AD and were associated with cognitive decline across the AD spectrum.

**Supplementary Information:**

The online version contains supplementary material available at 10.1186/s13195-022-01136-y.

## Introduction

Alzheimer’s disease (AD) is the most common cause of dementia and is becoming one of the most expensive and lethal diseases, resulting in a great social burden [1]. The pathophysiology of AD is mainly characterized by amyloid-β (Aβ) plaques, hyperphosphorylated tau, and neuroinflammation [1]. However, pharmacotherapies targeting Aβ and tau have achieved limited success [2]. Other upstream mechanisms, such as glymphatic dysfunction [3] and other clearance system damage, might also affect the clinical development of AD.

MRI-visible perivascular spaces (PVS) are fluid-filled spaces surrounding small penetrating blood vessels, which can be observed on brain MRI [4, 5]. Traditionally, MRI-visible PVS was regarded as an imaging marker of small vessel disease and correlated with aging [6]. However, the perivascular space has been increasingly suggested to play an important part in the glymphatic system, through which brain waste products are cleared from the brain [7, 8]. Impairment of the glymphatic system may lead to perivascular space enlargement and reduce the clearance of brain waste products, which would further lead to retrograde enlargement of the perivascular space [9]. Several studies have found associations between MRI-visible PVS and neurodegenerative diseases, including Parkinson’s disease [10–12], Huntington’s disease [13], frontotemporal lobar degeneration [14], and Fabry disease [15]. A previous study even found an association between MRI-visible PVS and brain tau deposition in a normal older population [16] and in military veterans with traumatic brain injury [17].

There have been cross-sectional studies with small samples (less than 200 subjects) investigating the relationship between MRI-visible PVS and AD [18–21]. However, the results of these studies were mixed, as some studies found more MRI-visible PVS in AD patients than in normal controls, while some studies did not. Furthermore, no specific study has investigated the role of MRI-visible PVS in predicting clinical progression and longitudinal cognitive change across the AD spectrum. In this longitudinal study, we aimed to investigate the distribution characteristics of MRI-visible PVS and the associations with cognitive decline and clinical progression across the AD spectrum.

## Materials and methods

### Study subjects

Data used in this study were obtained from the Alzheimer’s Disease Neuroimaging Initiative (ADNI) database, led by Principal Investigator Michael W. Weiner, M (http://adni.loni.usc.edu). The ADNI project was launched in 2003 and was designed to measure the progression of mild cognitive impairment and early Alzheimer’s disease by investigating serial MRI, PET, biological markers, and clinical and neuropsychological assessments. The ADNI study was approved by the institutional review boards of all participating institutions. Written informed consent was obtained from all the participants or their authorized representatives in accordance with the Declaration of Helsinki.

The specific enrolment procedure and inclusion criteria for the different diagnostic categories in the ADNI cohort have been described previously [22]. All subjects were classified as control (CN), mild cognitive impairment (MCI), or AD at each visit. CN subjects had normal cognitive function, with a Mini-Mental State Examination (MMSE) score of 24–30 and a clinical dementia rating (CDR) score of 0. MCI subjects had an MMSE score of 24–30, a global CDR of 0.5, and a memory box score of at least 0.5. AD subjects had an MMSE score of 20–26 and a global CDR≥0.5. Subjects diagnosed with EMCI or LMCI on the ADNI-2 were also considered to have MCI.

All study subjects who had a baseline brain MRI examination including T1-weighted, T2-weighted, and T2 fluid-attenuated inversion recovery (FLAIR) sequences were included from the ADNI database. The data from the ADNI-1 and ADNI-2 fulfilled these inclusion criteria and were used in this study. The time interval between MRI examination and baseline cognitive tests was within 1 month. We excluded 77 subjects with low MR image quality, and there was no significant difference between the included and excluded subjects in the CN, MCI, and AD groups (Supplementary Table 1). Finally, a total of 1429 individuals (486 CN, 667 MCI, and 276 AD) were included in this study.

### Clinical and cognitive assessments

We used multiple scales to assess cognitive functions, including global cognition by the 13-item AD Assessment Scale-cognitive subscale (ADAS-Cog 13) and MMSE. The ADNI memory composite score (ADNI-MEM), based on the Rey Auditory Verbal Learning task, word list learning and recognition tasks from ADAS-Cog, recall from Logical Memory I of the Wechsler Memory Test–Revised, and the 3-word recall item from the MMSE, was used to assess memory [23]. Executive function was assessed by the ADNI executive function score (ADNI-EF), which consisted of Category Fluency, Digit Span Backwards, Trail-Making Test Parts A and B, Wechsler Adult Intelligence Scale–Revised Digit–Symbol Substitution, and Clock Drawing items [24]. The baseline and the longest follow-up cognitive test data were collected.

Participant status at each visit was recorded as stable, reverted, or converted, with the former two statuses considered no progression and the latter considered progression, including CN conversion to MCI/AD and MCI conversion to AD. The last visit was used to determine the final diagnosis status for survival analysis. Notably, the definition of clinical progression was strictly defined beforehand in our study. For subjects who first progressed to MCI/AD and then reverted to their previous status, the first conversion to MCI/AD was not considered an event, as there may have been a mistake in the evaluation of the participant status. The progression time was calculated as the interval between the participant’s visit date when the conversion occurred and the baseline visit data.

### MRI acquisition and analysis

MR examinations were performed according to the ADNI MRI scanning protocol. Two trained neuroradiologists (M.L.W. and X.E.W. with 10 and 15 years of experience, respectively) who were blinded to the clinical diagnosis, demographic characteristics, and cognitive test results assessed the MR images according to the Standards for Reporting Vascular Changes on Neuroimaging (STRIVE) [25].

**Fig. 1:**
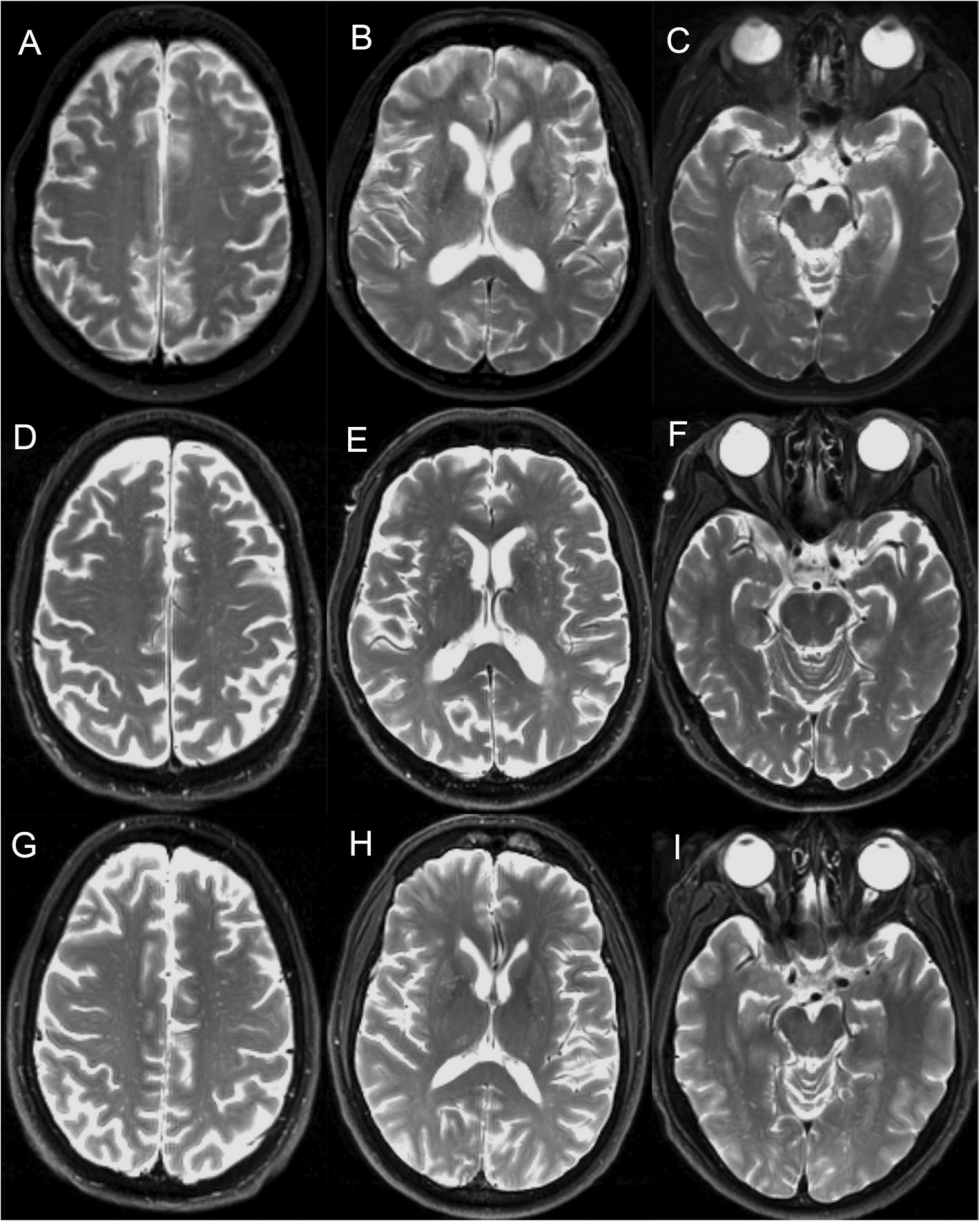
Examples of MRI-visible PVS across the AD spectrum. **A**–**C** A female control subject aged 78 had mild grade CSO-PVS, mild grade BG-PVS, and frequent/severe grade HP-PVS. After a follow-up of 24 months, this subject did not convert into MCI or AD. **D**–**F** A male MCI subject aged 75 had frequent/severe grade CSO-PVS, frequent/severe grade BG-PVS, and none HP-PVS. After a follow-up of 54 months, this subject converted into AD. **G**–**I** A male AD subject aged 62 had frequent/severe grade CSO-PVS, moderate grade BG-PVS, and mild grade HP-PVS. CN control, MCI mild cognitive impairment, AD Alzheimer disease, CSO centrum semiovale, BG basal ganglia, HP hippocampus, PVS perivascular spaces

The T1-weighted, T2-weighted, and T2 FLAIR sequences of the baseline MR images were comprehensively assessed for the existence of PVS, and the T2-weighted sequence was specifically used for counting PVS. MRI-visible PVS were located along the penetrating arteries and were characterized as round, ovoid, or linear lesions with a high signal on T2-weighted images and low signals on T1-weighted and FLAIR images (Fig. 1). PVS number was evaluated in the centrum semiovale (CSO), basal ganglia (BG), and hippocampus (HP). Furthermore, a validated 5-point visual rating scale was used to evaluate PVS severity (0 = no PVS, 1 = 1–10 PVS, 2 = 11–20 PVS, 3 = 21–40 PVS, and 4 = more than 40 PVS) for CSO-PVS and BG-PVS [26, 27]. Categories 0 and 1 were collapsed to no/mild severity; category 2 was classified as moderate severity; and categories 3 and 4 were classified as frequent/severe level. HP-PVS were subdivided by the number of PVS into no/mild severity (0–1 PVS), moderate severity (2–4 PVS), and frequent/severe level (>4 PVS) [21] (Fig. 1). White matter hyperintensity (WMH) volume and total intracranial volume were obtained; the method is described on the ADNI website (http://adni.loni.usc.edu, “4-Tissue Segmentation Methods for ADNI MR Scans.pdf”). The WMH volumes were nonnormally distributed and thus were log-transformed for analysis. The hippocampus volume was also extracted using the FreeSurfer method (http://adni.loni.usc.edu, “UCSF FreeSurfer Methods.pdf”).

The interrater reliability was excellent for the CSO-PVS number [intraclass correlation coefficient (ICC) = 0.936, 95% CI: 0.929–0.942], BG-PVS number (ICC = 0.903, 95% CI: 0.893–0.912), and HP-PVS number (ICC = 0.912, 95% CI: 0.903–0.921). The intra-rater reliability was determined from a random sample of 100 subjects with a 1-month interval between the first and second image assessments performed by the senior neuroradiologist. The intra-rater reliability was also excellent for the CSO-PVS number (ICC = 0.942, 95% CI: 0.915–0.961), BG-PVS number (ICC = 0.928, 95% CI: 0.895–0.951), and HP-PVS number (ICC = 0.920, 95% CI: 0.884–0.946). The PVS rating scale of the senior radiologist was used for the analysis.

### Statistical analysis

All statistical tests were performed by IBM SPSS Statistics for Windows, version 20.0, and R software (version 3.3.0; Vienna, Austria). Categorical variables were expressed as numbers (percentages) and were compared using the chi-square test among the CN, MCI, and AD groups. WMH and follow-up months were nonnormally distributed and thus were expressed as median [interquartile range (IQR)]. WMH volume was also log-transformed for further analysis. Other quantitative variables, including age, education, cognitive test scores, PVS number, HP volume, and intracranial volume, were expressed as the mean (standard deviation). Analyses of variance (ANOVAs) were used in the comparisons of all quantitative variables among the CN, MCI, and AD groups. Post hoc multiple comparisons were performed with Bonferroni tests.

The Kaplan–Meier method plotted with log-rank tests was used to investigate the association between MRI-visible PVS and clinical progression in the combined CN and MCI group. A Cox proportional hazard regression model was further conducted to estimate the hazard ratio (HR) with a 95% confidence intervals (CIs), adjusted by age, sex, education, APOɛ4, hypertension, diabetes, hyperlipidemia, coronary heart disease, atrial fibrillation, smoking, WMH volume, hippocampus volume, and intracranial volume. The three-level ordinal form of MRI-visible PVS was used. The no/mild severity group was set as the reference group, and the other groups were dummy coded for the survival analyses. The Cox proportional hazards assumption was assessed with the Schoenfeld residuals test, and all of the factors satisfied the proportional hazards hypothesis (*p*>0.05 for all). Individuals who did not develop MCI/AD were censored at the time of their last evaluation. These analyses were also performed for the CN and MCI groups separately.

Multivariable linear regression analyses were used to estimate the association between MRI-visible PVS number and baseline cognitive test performance. Linearity, normality, independence, and variance homogeneity were all fulfilled for the multivariable linear regression. To examine clinical progression in detail, we performed linear mixed-effects (LME) models with ADAS-Cog 13, MMSE, ADNI-MEM, and ADNI-EF as outcome variables and PVS number, time, and PVS number × time as predictors. The interaction term PVS number × time was the effect of interest, as it reflected whether MRI-visible PVS number moderated the relationship between time and cognitive performance. Our models contained random intercepts and slopes. Normality was fulfilled for the LME models. All the regression models included the following covariates: age, sex, education, APOɛ4, hypertension, diabetes, hyperlipidemia, coronary heart disease, atrial fibrillation, smoking, WMH volume, hippocampus volume, and intracranial volume, as these covariates may affect cognitive changes. Continuous independent variables and covariates in the models were *z*-scored prior to analysis for standardization. The regression model results are shown as beta coefficients, 95% CIs, and *p* values, and a forest plot was created for the whole-group LME model. *p* <0.05 derived from two-tailed tests was considered statistically significant.

## Results

### Participant characteristics

Participant demographics and cognitive performance are shown in Table 1. Compared with CN subjects, MCI subjects were younger (*p* = 0.017). There were fewer female subjects in the MCI group than in the CN group (*p* < 0.001). The MCI and AD groups were less educated and had more APOE ɛ4 carriers than the CN group (*p* < 0.001 for all). There was no significant difference in smoking status or cardiovascular disease history (*p* > 0.05 for all). On all cognitive measures, there were significant baseline differences between disease stages (*p* < 0.001 for all). After 36 (median) (IQR, 24–60) months of follow-up, 77 CN subjects (16.3%) converted to MCI status, and 10 CN subjects (2.1%) converted to AD status. In the MCI group, 273 subjects (42.5%) converted to AD status after a follow-up of 27 (median) (IQR, 18–36) months.

**Table 1:** Demographics and clinical characteristics across the Alzheimer’s disease spectrum

Characteristics	Data available	CN	Data available	MCI	Data available	AD	***p*** value
Age (years)	486	75.0 (5.8)	667	74.0 (7.4)	276	75.1 (7.8)	0.017*^,a^
Sex, female *n* (%)	486	251 (51.6)	667	263 (39.4)	276	127 (46.0)	<0.001*^,a^
Education (years)	486	16.3 (2.7)	667	16.0 (2.9)	276	15.1 (3.0)	<0.001*^,a,b,c^
APOE ɛ4 carriers *n* (%)	486	140 (28.8)	667	348 (52.2)	276	186 (67.4)	<0.001*^,a,b,c^
Hypertension, *n* (%)	486	238 (49.0)	667	331 (49.6)	276	138 (50.0)	0.958
Diabetes mellitus, *n* (%)	486	39 (8.0)	667	56 (8.4)	276	19 (6.9)	0.737
Hyperlipidemia, *n* (%)	486	215 (44.2)	667	306 (45.9)	276	134 (48.6)	0.517
Coronary heart disease, *n* (%)	486	30 (6.2)	667	44 (6.6)	276	14 (5.1)	0.675
Atrial fibrillation, *n* (%)	486	21 (4.3)	667	25 (3.7)	276	9 (3.3)	0.752
Smoking, *n* (%)	486	194 (39.9)	667	265 (39.7)	276	100 (36.2)	0.549
**Cognition**							
Baseline ADAS-Cog 13	484	9.21 (4.31)	657	17.26 (6.69)	261	29.53 (7.91)	<0.001*^,a,b,c^
Baseline MMSE score	486	29.0 (1.18)	666	27.1 (2.14)	276	22.9 (2.99)	<0.001*^,a,b,c^
Baseline ADNI-MEM score	483	1.07 (0.59)	656	0.069 (0.77)	265	−0.87 (0.57)	<0.001*^,a,b,c^
Baseline ADNI-EF score	483	0.77 (0.83)	656	0.12 (0.96)	265	−0.96 (0.94)	<0.001*^,a,b,c^
**Clinical progression**	472		642				<0.001*
Follow-up, months		36 (24, 60)		27 (18, 36)			
Conversion to MCI, *n* (%)		77 (16.3)					
Conversion to AD, *n* (%)		10 (2.1)		273 (42.5)			

Values are reported as mean (standard deviation) for the quantitative variables except follow-up month which is reported as median (interquartile range). Categorical variables are reported as frequency (percentage). *p<0.05. Group comparisons were done with the one-way ANOVA (quantitative variables) and chi-square test (categorical variables). ^a^*p* < 0.05 between CN vs MCI. ^b^*p* < 0.05 between CN vs AD. ^c^*p* < 0.05 between MCI vs AD. CN control, MCI mild cognitive impairment, AD Alzheimer disease, ADAS-Cog 13 13-item Alzheimer’s Disease Assessment Scale-cognitive subscale, MMSE Mini-Mental State Examination, ADNI-MEM ADNI memory composite score, ADNI-EF ADNI executive function score, ADNI Alzheimer’s Disease Neuroimaging Initiative

### Group comparisons and distribution characteristics of MRI-visible PVS across the AD spectrum

Table 2 shows the group comparisons and distribution characteristics of MRI-visible PVS and other neuroimaging findings across the AD spectrum. Compared with CN subjects, MCI and AD subjects had more CSO-PVS, both in number (*p* < 0.001) and grade (*p* < 0.001). However, there was no significant difference in BG-PVS and HP-PVS across the AD spectrum (*p* > 0.05 for all). Furthermore, MCI and AD subjects had smaller hippocampal volumes than CN subjects (*p* < 0.001). Compared with CN subjects, MCI subjects had a larger intracranial volume (*p* = 0.005). There was no significant difference in WMH volume (*p* > 0.05).

**Table 2:** Group comparisons of MRI-visible PVS and other MRI findings across the Alzheimer’s disease spectrum

	Data available	CN	Data available	MCI	Data available	AD	***p*** value
**CSO-PVS grade**	486		667		276		<0.001*^,a,b,c^
No/mild (grades 0–1), *n* (%)		125 (25.7)		120 (18.0)		32 (11.6)	
Moderate (grade 2), *n* (%)		239 (49.2)		326 (48.9)		126 (45.7)	
Frequent/severe (grades 3–4), *n* (%)		122 (25.1)		221 (33.1)		118 (42.8)	
CSO-PVS number		15.9 (7.9)		17.5 (8.4)		19.0 (8.3)	<0.001*^,a,b,c^
**BG-PVS grade**	486		667		276		0.183
No/mild (grades 0–1), *n* (%)		399 (82.1)		531 (79.6)		221 (80.1)	
Moderate (grade 2), *n* (%)		78 (16.0)		126 (18.9)		45 (16.3)	
Frequent/severe (grades 3–4), *n* (%)		9 (1.9)		10 (1.5)		10 (3.6)	
BG-PVS number		7.2 (4.1)		7.4 (4.1)		7.7 (4.8)	0.326
**HP-PVS grade**	486		667		276		0.432
No/mild (0–1 PVS), *n* (%)		137 (28.2)		200 (30.0)		93 (33.7)	
Moderate (2–4 PVS), *n* (%)		217 (44.7)		290 (43.5)		122 (44.2)	
Frequent/severe (>4 PVS), *n* (%)		132 (27.2)		177 (26.5)		61 (22.1)	
HP-PVS number		3.2 (2.5)		3.0 (2.5)		2.8 (2.3)	0.182
WMH volume (cm^3^)	486	1.25 (0.25, 3.91)	666	0.79 (0.20, 3.28)	275	1.14 (0.24, 3.93)	0.057
HP volume (cm^3^)	483	7.33 (0.94)	653	6.57 (1.15)	262	5.60 (1.03)	<0.001*^,a,b,c^
Intracranial volume, cm^3^	486	1415 (138)	665	1442 (145)	276	1420 (157)	0.005*^,a^

Values are reported as mean (standard deviation) for the quantitative variables except WMH volume which is reported as median (interquartile range). *p<0.05. Group comparisons were done with the one-way ANOVA (quantitative variables) and chi-square test (categorical variables). WMH volume was log-transformed for one-way ANOVA analysis. ^a^*p* < 0.05 between CN vs MCI. ^b^*p* < 0.05 between CN vs AD. ^c^*p* < 0.05 between MCI vs AD. CN control, MCI mild cognitive impairment, AD Alzheimer disease, CSO centrum semiovale, BG basal ganglia, HP hippocampus, PVS perivascular spaces, WMH white matter hyperintensities

### MRI-visible PVS and risks of diagnostic conversion into MCI/AD

**Fig. 2:**
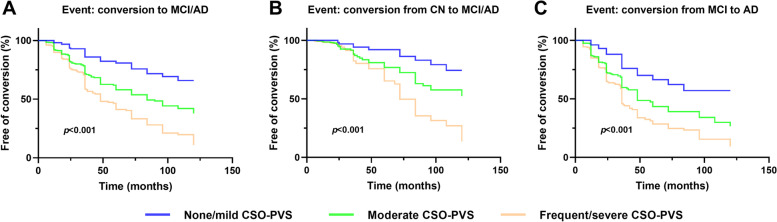
Kaplan–Meier curves showing survival probability of clinical progression according to different CSO-PVS levels. **A** Comparison of free of conversion to MCI/AD rate in combined CN and MCI subjects according to different CSO-PVS levels. **B** Comparison of free of conversion to MCI/AD rate in CN subjects according to different CSO-PVS levels. **C** Comparison of free of conversion to AD rate in MCI subjects according to different CSO-PVS levels. MCI mild cognitive impairment, AD Alzheimer disease, CSO centrum semiovale, PVS perivascular spaces

The Kaplan–Meier curves of diagnostic conversion according to the grades of CSO-PVS are shown in Fig. 2. Individuals with moderate and frequent/severe grade CSO-PVS had a higher diagnostic conversion risk than individuals with no/mild grade CSO-PVS (log-rank *p* < 0.001 for all) in the combined CN and MCI group. When stratified into separate CN and MCI groups, the risk difference of diagnostic conversion among CSO-PVS grades remained significant (log-rank *p* < 0.001 for all).

Further Cox regression analyses revealed that moderate and frequent/severe CSO-PVS were associated with a higher risk of diagnostic conversion (HR = 2.007, 95% CI = 1.382–2.914, *p* < 0.001; HR = 2.676, 95% CI = 1.830–3.911, *p* < 0.001, respectively) in the combined CN and MCI group. In the CN group, individuals with moderate and frequent/severe CSO-PVS had a higher progression rate to MCI/AD (HR = 2.023, 95% CI = 1.033–3.963, *p* = 0.040; HR = 3.504, 95% CI = 1.728–7.106, *p* < 0.001, respectively). In the MCI group, individuals with moderate and frequent/severe CSO-PVS had a higher progression rate to AD (HR = 1.878, 95% CI = 1.197–2.947, *p* = 0.006; HR = 2.303, 95% CI = 1.462–3.628, *p* < 0.001, respectively).

### Cross-sectional and longitudinal association between CSO-PVS and cognitive measures

Table 3 shows the cross-sectional and longitudinal association between CSO-PVS and cognitive measures. In the cross-sectional analysis, a higher CSO-PVS number was positively associated with ADAS-Cog 13 (*β* = 0.086, *p* < 0.001; *β* = 0.060, *p* = 0.014) and negatively associated with MMSE (*β* = −0.161, *p* < 0.001; *β* = −0.151, *p* < 0.001), ADNI-MEM (*β* = −0.093, *p* < 0.001; *β* = −0.060, *p* = 0.023), and ADNI-EF (*β* = −0.124, *p* < 0.001; *β* = −0.134, *p* < 0.001) in all subjects and in the MCI group. The association remained significant with ADNI-EF in the CN group (*β* = −0.128, *p* < 0.001) and MMSE in the AD group (*β* = −0.190, *p* < 0.003). The detailed beta coefficients for all variables in the regression models can be found in Supplementary Tables 2, 3, 4 and 5.

**Table 3:** Cross-sectional and longitudinal multiple linear regression of CSO-PVS with cognitive performance

	Total		CN		MCI		AD	
	***β*** (95% CI)	***p*** value	***β*** (95% CI)	***p*** value	***β*** (95% CI)	***p*** value	***β*** (95% CI)	***p*** value
**Baseline**								
ADAS-Cog 13	0.086 (0.044, 0.127)	<0.001*	0.009 (−0.033, 0.050)	0.677	0.060 (0.012, 0.107)	0.014*	0.042 (−0.063, 0.146)	0.432
MMSE	−0.161 (−0.205, −0.116)	<0.001*	−0.035 (−0.072, 0.002)	0.063	−0.151 (−0.201, −0.101)	<0.001*	−0.190 (−0.314, −0.065)	0.003*
ADNI-MEM	−0.093 (−0.133, −0.053)	<0.001*	−0.053 (−0.107, 0.001)	0.053	−0.060 (−0.112, −0.008)	0.023*	−0.020 (−0.092, 0.052)	0.585
ADNI-EF	−0.124 (−0.170, −0.077)	<0.001*	−0.128 (−0.193, −0.062)	<0.001*	−0.134 (−0.197, −0.071)	<0.001*	0.074 (−0.036, 0.185)	0.187
**LME model**								
ADAS-Cog 13	0.060 (0.026, 0.094)	0.001*	0.025 (0.002, 0.048)	0.032*	0.094 (0.046, 0.142)	<0.001*	0.015 (−0.164, 0.194)	0.870
MMSE	−0.069 (−0.108, −0.030)	0.001*	−0.054 (−0.081, −0.026)	<0.001*	−0.106 (−0.169, −0.043)	0.001*	0.287 (0.014, 0.559)	0.039*
ADNI-MEM	−0.058 (−0.090, −0.026 )	<0.001*	−0.073 (−0.109, −0.037)	<0.001*	−0.043 (−0.092, 0.006)	0.085	0.018 (−0.126, 0.162)	0.805
ADNI-EF	−0.048 (−0.083, −0.012)	0.009*	−0.042 (−0.079, −0.006)	0.023*	−0.020 (−0.075, 0.035)	0.473	−0.088 (−0.308, 0.132)	0.432

Models were adjusted for age, sex, education, apo ɛ4, hypertension, diabetes, hyperlipidemia, coronary heart disease, atrial fibrillation, smoking, WMH volume, hippocampus volume, and intracranial volume. Continuous independent variables and covariates in the models were *z*-scored prior to analysis for standardization. In the LME model, CSO-PVS*time was the effect of interest, as it reflected whether CSO-PVS moderated the relationship between time and cognitive decline. *p < 0.0.5. CSO centrum semiovale, PVS perivascular spaces, CN control, MCI mild cognitive impairment, AD Alzheimer disease, ADAS-Cog 13 13-item Alzheimer’s Disease Assessment Scale-cognitive subscale, MMSE Mini-Mental State Examination, ADNI-MEM ADNI memory composite score, ADNI-EF ADNI executive function score, ADNI Alzheimer’s Disease Neuroimaging Initiative, LME linear mixed-effects model

**Fig. 3:**
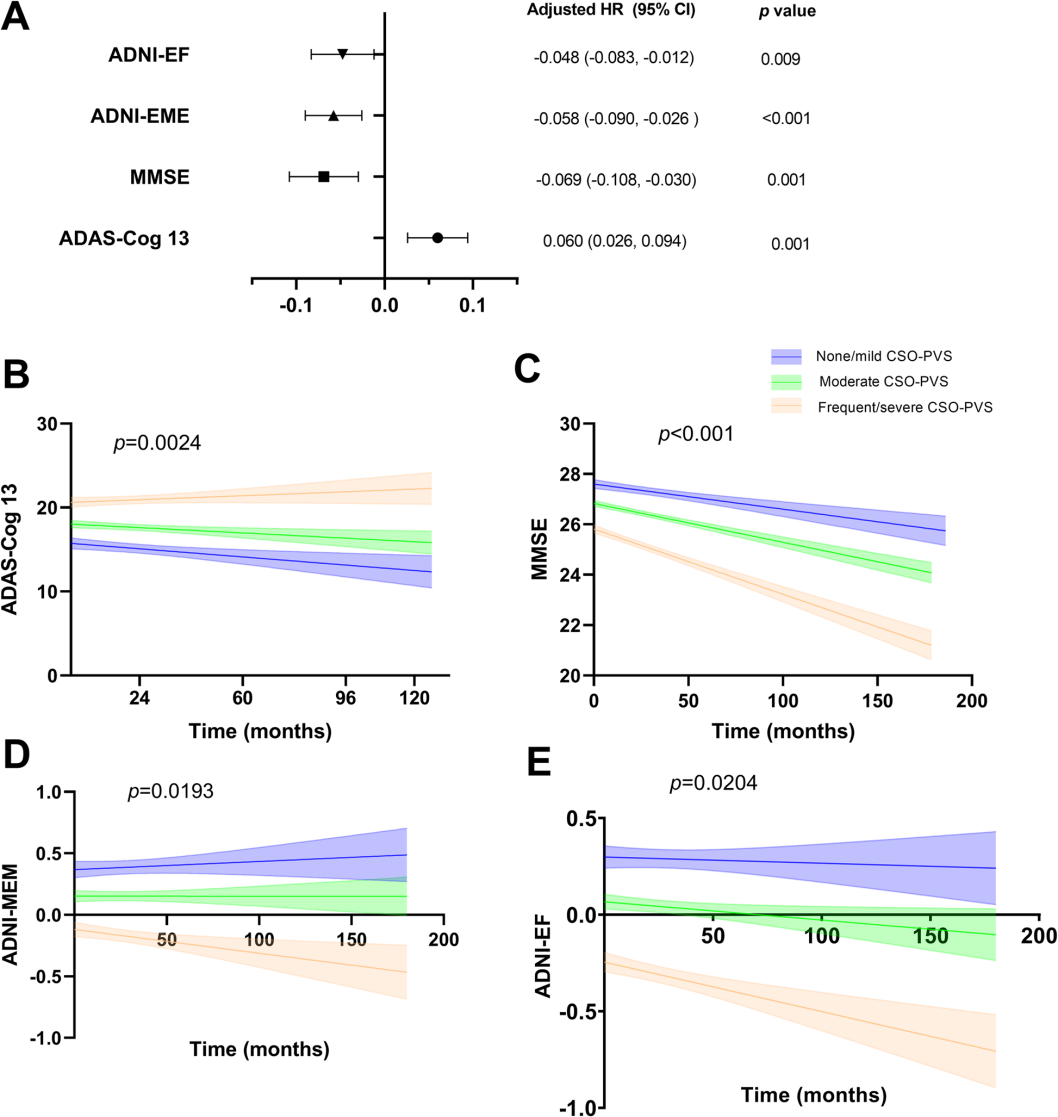
Changes in longitudinal cognitive performance by level of CSO-PVS. **A** Cognitive performance among different levels of CSO-PVS groups based on the linear mixed-effects model adjusting for age, sex, education, apo ɛ4, hypertension, diabetes, hyperlipidemia, coronary heart disease, atrial fibrillation, smoking, WMH volume, hippocampus volume, and intracranial volume. **B**–**E** Trajectories of estimated cognitive performance in ADAS-Cog 13, MMSE, ADNI-MEM, and ADNI-EF by level of CSO-PVS. ADAS-Cog 13 13-item Alzheimer’s Disease Assessment Scale-cognitive subscale, MMSE Mini-Mental State Examination, ADNI-MEM ADNI memory composite score, ADNI-EF ADNI executive function score, ADNI Alzheimer’s Disease Neuroimaging Initiative

LME models showed that a higher CSO-PVS number was associated with cognitive decline in the ADAS-Cog 13 (*β* = 0.060, *p* = 0.001; *β* = 0.025, *p* = 0.032), MMSE (*β* = −0.069, *p* =0.001; *β* = −0.054, *p* < 0.001), ADNI-MEM (*β* = −0.058, *p* < 0.001; *β* = −0.073, *p* < 0.001), and ADNI-EF (*β* = −0.048, *p* = 0.009; *β* = −0.042, *p* = 0.023) in all subjects and in the CN group. The association remained significant with ADAS-Cog 13 (*β* = 0.094, *p* < 0.001) and MMSE (*β* = −0.106, *p* = 0.001) scores in the MCI group and MMSE scores in the AD group (*β* = 0.287, *p* = 0.039). The detailed beta coefficients for all the variables in the regression models can be found in Supplementary Tables 6, 7, 8 and 9. As shown in Fig. 3, individuals with moderate and frequent/severe CSO-PVS had a more rapid decline in all cognitive measures than subjects with no/mild CSO-PVS.

## Discussion

In this study, we investigated the characteristics and associations of MRI-visible PVS with clinical progression and longitudinal cognitive decline across the AD spectrum. Our study found that MCI and AD subjects had more CSO-PVS than CN subjects. Individuals with moderate and frequent/severe grade CSO-PVS had a more than 2-fold higher clinical progression risk than individuals with no/mild grade CSO-PVS. A higher CSO-PVS number was associated with baseline cognitive performance and longitudinal cognitive decline in all cognitive tests.

With the wide use of MRI, MRI-visible PVS are frequently detected in clinical practice. Emerging literature suggests that arterial stiffening and abnormal protein aggregation in vessel walls are two potential mechanisms that drive PVS formation [28]. Arterial stiffening, influenced by age, hypertension, and other cardiovascular diseases, was shown to have an association with BG-PVS [29–31]. Notably, CSO-PVS and HP-PVS may also be affected by cardiovascular risk factors and cerebral small vessel disease [32, 33]. Regarding abnormal protein aggregation, CSO-PVS was proven to be most commonly affected in cerebral amyloid angiopathy (CAA) with Aβ deposition [34].

In our study, compared with CN subjects, MCI and AD subjects had more CSO-PVS both in number and grade. This was similar to previous studies [18–20]. Notably, a recent study using a quantitative method also revealed that MCI subjects had more CSO-PVS than normal controls [35]. A high degree of CSO-PVS has been suggested to be associated with CAA, which is characterized by neurovascular accumulation of Aβ and deficiencies in the clearance of Aβ along the arterial perivascular space [36, 37]. A postmortem AD sample study showed increased PVS and global AQP4 expression with a qualitative reduction in perivascular localization of AQP4 in frontal cortical tissue compared with control subjects, and increasing PVS burden was associated with the presence of tau and Aβ pathology [38]. As AQP4 has a clear role in brain glymphatic pathway function [39], PVS may reflect failed glymphatic function leading to clearance deficiency in parenchymal Aβ and other brain waste in AD.

However, there was no significant difference in BG-PVS across the AD spectrum. BG-PVS was more related to hypertensive arteriopathy [40, 41] and vascular dementia [20, 42]. As there was no significant difference in cardiovascular disease history across the AD spectrum in our study, the effect of cardiovascular disease on BG-EPVS could be neglectable. Our study suggested no association between BG-PVS and the AD spectrum. Furthermore, we found no significant difference in HP-PVS among the AD spectrum. Previous studies suggested that age and hypertension were the two main factors associated with HP-PVS [32, 43, 44], and HP-PVS was not associated with the occurrence of dementia [32]. The development of MRI-visible HP-PVS may have little association with AD. However, a small sample size (39 AD patients) study found increased HP-PVS but no significant difference in CSO-PVS in AD [21]. The contradictory results may be caused by different study designs and enrolled populations. The enrolled population in their study tended to have more cardiovascular disease, which would have a nonnegligible effect on the occurrence of MRI-visible PVS.

Regarding the relationship between MRI-visible PVS and cognitive impairment, there were mixed findings. Two meta-analyses found no association of MRI-visible PVS with cognitive impairment cross-sectionally [45, 46]. However, a longitudinal study revealed that severe MRI-visible PVS, especially CSO-PVS, was associated with an increased risk of cognitive decline and dementia in community-dwelling older adults [47], and a recent study revealed that CSO-PVS was associated with the progression of cognitive decline in an amyloid-independent manner in the AD continuum [48]. In our study, CSO-PVS was also associated with baseline cognitive performance and longitudinal cognitive decline in all subjects after adjusting for cardiovascular disease and other risk factors, indicating that there was an independent association of CSO-PVS with cognitive impairment. As mentioned above, we speculated that CSO-PVS may represent impaired glymphatic function causing clearance deficiency of amyloid and other toxins, leading to cognitive impairment. However, some cognitive tests were not associated with CSO-PVS in the separate CN, MCI, and AD group analyses. We speculate that the weak associations may be caused by less statistical power in the separate group analysis, while the disease severity would mediate the relationship between CSO-PVS and cognition in the combined analysis. Future studies using more sensitive methods, such as quantitative CSO-PVS volume analyses in separate groups, are warranted to replicate our results.

Interestingly, individuals with moderate and frequent/severe grade CSO-PVS had a more than 2-fold higher diagnostic conversion risk than individuals with no/mild grade CSO-PVS in the CN and MCI groups. In the CN group, individuals with frequent/severe CSO-PVS even had a more than 3-fold higher progression rate to MCI/AD than individuals with no/mild grade CSO-PVS. To our knowledge, this was the first study to investigate the value of MRI-visible PVS in the diagnostic conversion risk across the AD spectrum. Our study suggested the role of CSO-PVS in the development of AD and provided a potential novel imaging biomarker for disease severity assessment.

Our study should be interpreted in light of the following limitations. First, the ADNI project had strict inclusion and exclusion criteria, and the enrolled subjects were not representative of the wider general population [49]. This may be considered an advantage, as the sample represented a relatively pure sample with little confounding. However, the results may not necessarily ensure generalizability. Second, Aβ and tau biomarkers were not investigated in the relationship between MRI-visible PVS and cognitive impairment. Aβ and tau may play an important role in the relationship. However, there were too many missing data about CSF or PET biomarkers of Aβ and tau. Future research on the role of Aβ and tau in the relationship between MRI-visible PVS and cognitive impairment is warranted. Third, PVS in the hippocampus are very difficult to distinguish from hippocampal sulcus remnant cysts [50]. The results of MRI-visible PVS in the hippocampus should be interpreted with caution. Fourth, no quantitative analysis was performed to assess PVS, and we only used validated visual rating scales, which were rater-dependent and had ceiling or floor effects. However, the assessment method was simple, and the interrater and intra-rater reliability for the rating of MRI-visible PVS was excellent. The visual rating method could be applied easily in clinical practice.

In conclusion, our study demonstrated that CSO-PVS were more common in MCI and AD. CSO-PVS were associated with cognitive decline across the AD spectrum. Our study suggested that MRI-visible PVS could be a novel imaging assessment biomarker and a new potential therapeutic target for cognitive decline in the AD spectrum.

## Supplementary Information

**Additional file 1: Supplementary table 1.** Demographics and Clinical Characteristics across the Alzheimer's disease spectrum between included and excluded subjects.

**Additional file 2: Supplementary table 2.** Cross-sectional multivariable linear regression of CSO-PVS with ADAS-Cog 13 across the Alzheimer's disease spectrum.

**Additional file 3: Supplementary table 3.** Cross-sectional multivariable linear regression of CSO-PVS with MMSE across the Alzheimer's disease spectrum.

**Additional file 4: Supplementary table 4.** Cross-sectional multivariable linear regression of CSO-PVS with ADNI-MEM across the Alzheimer's disease spectrum.

**Additional file 5: Supplementary table 5.** Cross-sectional multivariable linear regression of CSO-PVS with ADNI-EF across the Alzheimer's disease spectrum.

**Additional file 6: Supplementary table 6.** Longitudinal linear mixed-effects regression of CSO-PVS with ADAS-13 across the Alzheimer's disease spectrum.

**Additional file 7: Supplementary table 7.** Longitudinal linear mixed-effects regression of CSO-PVS with MMSE across the Alzheimer's disease spectrum.

**Additional file 8: Supplementary table 8.** Longitudinal linear mixed-effects regression of CSO-PVS with ADNI-MEM across the Alzheimer's disease spectrum.

**Additional file 9: Supplementary table 9.** Longitudinal linear mixed-effects regression of CSO-PVS with ADNI-EF across the Alzheimer's disease spectrum.

## Data Availability

All imaging, demographics, and neuropsychological data used in this article are publicly available and were downloaded from the ADNI website (adni.loni.usc.edu). Upon request, we will provide a list of ADNI participant identifications for replication purposes.
