# Supplementary material for: Associations of MRI-visible perivascular spaces with longitudinal cognitive decline across the Alzheimer’s disease spectrum
Source: Alzheimers Res Ther. 2022 Dec 13;14:185. doi: 10.1186/s13195-022-01136-y (PMC9746143; doi:10.1186/s13195-022-01136-y)
Supplement: Supplementary file 1 — Additional file 1: Supplementary table 1. Demographics and Clinical Characteristics across the Alzheimer's disease spectrum between included and excluded subjects. [file 13195_2022_1136_MOESM1_ESM.docx]

**Supplementary table 1 Demographics and Clinical Characteristics across the Alzheimer's disease spectrum between included and excluded subjects**

| **Characteristics** | **Included CN n=486** | **Excluded**  **CN n=12** | ***p* value** | **Included MCI n=667** | **Excluded**  **MCI n=32** | ***p* value** | **Included AD n=276** | **Excluded**  **AD n=33** | ***p* value** |
| --- | --- | --- | --- | --- | --- | --- | --- | --- | --- |
| Age (years) | 75.0 (5.8) | 72.5 (5.7) | 0.141 | 74.0 (7.4) | 75.7 (7.4) | 0.205 | 75.1 (7.8) | 78.2 (7.0) | **0.029*** |
| Sex, female n (%） | 251 (51.6) | 5 (41.7) | 0.494 | 263 (39.4) | 12 (37.5) | 0.827 | 127 (46.0) | 13 (39.4) | 0.470 |
| Education (years) | 16.3 (2.7) | 17.0 (2.6) | 0.404 | 16.0 (2.9) | 15.4 (2.6) | 0.304 | 15.1 (3.0) | 15.6 (2.9) | 0.367 |
| APOE ɛ4 carriers n (%）^a^ | 140 (28.8) | 0 (0) | 0.293 | 348 (52.2) | 18(56.3) | 0.652 | 186 (67.4) | 19 (65.5) | 0.838 |
| Hypertension, n (%) | 238 (49.0) | 4 (33.3) | 0.284 | 331 (49.6) | 17 (53.1) | 0.699 | 138 (50.0) | 19 (57.6) | 0.411 |
| Diabetes mellitus, n (%) | 39 (8.0) | 2 (16.7) | 0.586 | 56 (8.4) | 7 (21.9) | **0.009*** | 19 (6.9) | 6 (18.2) | **0.024*** |
| Hyperlipidemia, n (%) | 215 (44.2) | 4 (33.3) | 0.647 | 306 (45.9) | 14 (43.8) | 0.813 | 134 (48.6) | 18 (54.5) | 0.515 |
| Coronary heart disease, n (%) | 30 (6.2) | 0 (0) | 0.784 | 44 (6.6) | 2 (6.3) | 1.000 | 14 (5.1) | 3 (9.1) | 0.580 |
| Atrial fibrillation, n (%) | 21 (4.3) | 1 (8.3) | 1.000 | 25 (3.7) | 2 (6.3) | 0.804 | 9 (3.3) | 0 (0) | 0.613 |
| Smoking, n (%) | 194 (39.9) | 5 (41.7) | 0.903 | 265 (39.7) | 16 (50.0) | 0.247 | 100 (36.2) | 15 (45.5) | 0.300 |
| **Cognition ^b^** |  |  |  |  |  |  |  |  |  |
| Baseline ADAS-Cog 13 | 9.21 (4.31) | 9.05 (4.70) | 0.920 | 17.26 (6.69) | 19.81 (7.60) | 0.076 | 29.53 (7.91) | 30.01 (6.63) | 0.749 |
| Baseline MMSE score | 29.0 (1.18) | 28.6 (1.78) | 0.202 | 27.1 (2.14) | 26.9 (2.15) | 0.680 | 22.9 (2.99) | 21.8 (3.10) | 0.058 |
| Baseline ADNI-MEM score | 1.07 (0.59) | 1.07 (0.56) | 0.990 | 0.069 (0.77) | -0.080 (0.77) | 0.295 | -0.87 (0.57) | -0.99 (0.56) | 0.270 |
| Baseline ADNI-EF score | 0.77 (0.83) | 0.48 (0.73) | 0.363 | 0.12 (0.96) | -0.033 (0.96) | 0.392 | -0.96 (0.94) | -1.19 (0.92) | 0.215 |

Values are reported as mean (standard deviation) for the quantitative variables and as frequency (percentage) for the categorical variables. * *p*<0.05. Group comparisons were done with the t-test (quantitative variables) and chi-square test (categorical variables). a APOE ɛ4 was missing in 5 excluded CN subjects and 4 excluded AD subjects. b Baseline ADAS-Cog 13 was missing in 2 CN, 10 MCI, and 5 AD included subjects. Baseline MMSE score was missing in 1 included MCI subjects. Baseline ADNI-MEM score was missing in 3 CN, 11 MCI, and 11AD included subjects. Baseline ADNI-EF was missing in 3 CN, 11 MCI, and 11 AD included subjects. The baseline ADAS-Cog 13, MMSE, ADNI-MEM and ADNI-EF were missing in 5 CN, 1MCI, and 3 AD excluded subjects. CN = Control; MCI = mild cognitive impairment; AD = Alzheimer disease; ADAS-Cog 13 = 13-item Alzheimer’s Disease Assessment Scale-cognitive subscale; MMSE = Mini-Mental State Examination; ADNI-MEM = ADNI memory composite score; ADNI-EF = ADNI executive function score; ADNI = Alzheimer’s Disease Neuroimaging Initiative.
