# Supplementary material for: Associations of MRI-visible perivascular spaces with longitudinal cognitive decline across the Alzheimer’s disease spectrum
Source: Alzheimers Res Ther. 2022 Dec 13;14:185. doi: 10.1186/s13195-022-01136-y (PMC9746143; doi:10.1186/s13195-022-01136-y)
Supplement: Supplementary file 8 — Additional file 8: Supplementary table 8. Longitudinal linear mixed-effects regression of CSO-PVS with ADNI-MEM across the Alzheimer's disease spectrum. [file 13195_2022_1136_MOESM8_ESM.docx]

**Supplementary table 8 Longitudinal linear mixed-effects regression of CSO-PVS with ADNI-MEM across the Alzheimer's disease spectrum**

|  | **Total** | | **CN** | | **MCI** | | **AD** | |
| --- | --- | --- | --- | --- | --- | --- | --- | --- |
|  | **β (95% CI)** | ***p*-Value** | **β (95% CI)** | ***p*-Value** | **β (95% CI)** | ***p*-Value** | **β (95% CI)** | ***p*-Value** |
| Intercept | -0.045(-0.252,0.161) | 0.667 | 0.607(-0.383,1.597) | 1.000 | -0.1(-1.637,1.437) | 0.899 | -0.617(-1.501,0.266) | 0.171 |
| Age | 0.111(0.078,0.143) | **<0.001*** | -0.126(-0.178,-0.074) | **<0.001*** | 0.03(-0.013,0.073) | 0.175 | 0.069(0.019,0.119) | **0.007*** |
| Sex | -0.284(-0.36,-0.209) | **<0.001*** | -0.308(-0.405,-0.211) | **<0.001*** | -0.145(-0.247,-0.042) | **0.006*** | -0.148(-0.282,-0.013) | **0.031*** |
| Education | 0.143(0.114,0.173) | **<0.001*** | 0.143(0.101,0.184) | **<0.001*** | 0.108(0.069,0.146) | **<0.001*** | 0.012(-0.039,0.062) | 0.652 |
| Apo ɛ4 | 0.301(0.241,0.361) | **<0.001*** | 0.083(-0.001,0.166) | 0.053 | 0.293(0.214,0.372) | **<0.001*** | 0.041(-0.061,0.143) | 0.427 |
| Hypertension | 0.05(-0.008,0.107) | 0.089 | 0.046(-0.03,0.123) | 0.235 | 0.055(-0.022,0.133) | 0.161 | 0.071(-0.027,0.168) | 0.155 |
| Diabetes | 0.053(-0.054,0.16) | 0.330 | 0.061(-0.086,0.207) | 0.416 | 0.046(-0.093,0.185) | 0.515 | -0.056(-0.244,0.131) | 0.555 |
| Hyperlipidemia | -0.03(-0.088,0.029) | 0.318 | -0.028(-0.106,0.05) | 0.486 | -0.031(-0.109,0.046) | 0.429 | -0.049(-0.145,0.047) | 0.318 |
| Coronary heart disease | 0.056(-0.063,0.176) | 0.354 | -0.019(-0.177,0.138) | 0.811 | 0.151(-0.003,0.305) | 0.055 | -0.246(-0.479,-0.012) | **0.04*** |
| Atrial fibrillation | -0.035(-0.184,0.114) | 0.643 | 0.119(-0.078,0.315) | 0.235 | -0.263(-0.466,-0.059) | **0.011*** | -0.062(-0.311,0.186) | 0.622 |
| Smoking | -0.019(-0.077,0.039) | 0.526 | 0.065(-0.011,0.141) | 0.095 | -0.016(-0.094,0.061) | 0.677 | -0.029(-0.131,0.073) | 0.576 |
| WMH volume | 0.008(-0.021,0.037) | 0.602 | -0.03(-0.06,0) | **0.048*** | 0.044(-0.003,0.091) | 0.069 | -0.062(-0.141,0.017) | 0.122 |
| Hippocampus volume | 0.576(0.543,0.609) | **<0.001*** | 0.135(0.075,0.195) | **<0.001*** | 0.432(0.385,0.48) | **<0.001*** | 0.191(0.124,0.259) | **<0.001*** |
| Intracranial volume | -0.164(-0.202,-0.126) | **<0.001*** | -0.066(-0.121,-0.012) | **0.018*** | -0.13(-0.18,-0.079) | **<0.001*** | -0.02(-0.081,0.042) | 0.530 |
| CSO-PVS | -0.111(-0.14,-0.081) | **<0.001*** | -0.08(-0.121,-0.04) | **<0.001*** | -0.08(-0.121,-0.04) | **<0.001*** | 0.003(-0.08,0.086) | 0.951 |
| Time | -0.125(-0.157,-0.094) | **<0.001*** | -0.146(-0.181,-0.111) | **<0.001*** | -0.22(-0.268,-0.173) | **<0.001*** | -0.512(-0.669,-0.355) | **<0.001*** |
| CSO-PVS*time | -0.058(-0.09,-0.026) | **<0.001*** | -0.073(-0.109,-0.037) | **<0.001*** | -0.043(-0.092,0.006) | 0.085 | 0.018(-0.126,0.162) | 0.805 |

Models were adjusted for age, sex, education, apo ɛ4, hypertension, diabetes, hyperlipidemia, coronary heart disease, atrial fibrillation, smoking, WMH volume, hippocampus volume and intracranial volume. Continuous independent variables and covariates in the models were z-scored prior to analysis for standardization. CSO-PVS*time was the effect of interest, as it reflected whether CSO-PVS moderated the relationship between time and ADNI-MEM change. *: *p* < 0.0.5. CSO = centrum semiovale; PVS = perivascular spaces; CN = Control; MCI = mild cognitive impairment; AD = Alzheimer disease; ADNI-MEM = ADNI memory composite score; ADNI = Alzheimer’s Disease Neuroimaging Initiative.
